# Genome-wide association study of coronary artery calcification in asymptomatic Korean populations

**DOI:** 10.1371/journal.pone.0214370

**Published:** 2019-03-28

**Authors:** Su-Yeon Choi, Eunsoon Shin, Eun Kyung Choe, Boram Park, Heesun Lee, Hyo Eun Park, Jong-Eun Lee, Seung Ho Choi

**Affiliations:** 1 Department of Internal Medicine, Seoul National University Hospital Healthcare System Gangnam Center, Seoul, Korea; 2 Department of Internal Medicine, Seoul National University College of Medicine, Seoul, Korea; 3 DNA Link, Inc., Seoul, Korea; 4 Department of Surgery, Seoul National University Hospital Healthcare System Gangnam Center, Seoul, Korea; 5 Department of Public Health Science, Seoul National University, Seoul, Korea; GeneDx, UNITED STATES

## Abstract

Epidemiologic evidence indicates that the prevalence and severity of coronary artery disease vary depending on ethnicity. In this study, a genome-wide association study for coronary artery calcification (CAC) was performed in a Korean population-based sample of 400 subjects without prior coronary artery disease and replicated in another of 1,288 subjects. CAC score, as assessed by multi-detector computed tomography, was evaluated in volunteers for screening purposes as part of a routine health examination. CAC score greater than the 90th percentile across the age in each sex group was considered severe CAC. Single nucleotide polymorphisms (SNPs) associated with severe CAC after adjusting for age, sex, hypertension, and diabetes were investigated using the additive model of logistic regression. One SNP (rs10757272 in the intronic region of the *CDKN2B-AS1* gene in chromosome 9p21.3) met Bonferroni correction in the discovery set (p = 7.55E-08) and was also significant in the validation set by TaqMan assay (p = 0.036). Subjects with rs10757272 were found to have an increased odds ratio (OR) of having severe CAC in multivariate logistic regression analysis after adjusting for age, sex, hypertension, and diabetes (adjusted OR 3.24 and 95% CI 2.11–4.97). In conclusion, SNP rs10757272 in chromosome 9p21.3 was associated with severe CAC based on age and sex in an asymptomatic community-based Korean population. Therefore, it was associated with promotion of coronary artery calcification in subclinical state.

## Introduction

Coronary artery calcium score (CACS) represents a measure of overall coronary atheromatous plaque burden and is a strong predictor of coronary artery disease (CAD). Epidemiological studies have identified that the development of coronary atherosclerosis is associated with many risk factors [1,2]. These factors include plasma lipid concentrations, blood pressure, smoking, diabetes, inflammation markers, and lifestyle. However, the role of traditional risk factors has been proven only for some. A twin and family study in the Swedish Twin Registry has estimated the total heritability of susceptibility to CAD to be a significant proportion (40–50%) [3]. Therefore, genetic analysis has the potential to identify pathways of and therapeutic targets against CAD. A recent study has provided insights into the genetic basis of CAD and identified key biological pathways [4]. Genetic factors are defined as an important risk contributor for the pathogenesis of CAD and myocardial infarction (MI) [5–10].

Epidemiologic evidence indicates that the prevalence and severity of CAD vary depending on ethnicity [11]. Moreover, ethnic disparity with regard to coronary artery calcification (CAC) has been reported. In asymptomatic individuals without known CAD, Korean adults have a lower prevalence and severity of CAC compared with American adults [12]. Meanwhile, African Americans are less likely to develop CAC and have markedly lower levels of CACS than European Americans despite exposure to more severe conventional CVD risk factors [13]. The precise mechanisms that might influence these ethnic disparities are not well understood, but genetic factors may explain this variability by providing the basis for differences in individual susceptibility to CAD [14–16].

Most genome-wide association studies (GWASs) have been performed mainly in populations of European descent. The exact genetic markers and mechanisms to influence the risk of CAD in Koreans remain largely unknown.

In this study, we aimed to identify single nucleotide polymorphisms (SNPs) that could be associated with severe CAC in asymptomatic Korean populations using a GWAS.

## Materials and methods

### Study design and population

After obtaining informed consent for blood storage, blood samples were collected from subjects during a routine health check-up program at the Seoul National University Hospital Healthcare System Gangnam Center from January 2014 to March 2016. Blood samples were then stored at a biorepository. Among the subjects, asymptomatic subjects (n = 2,188) who had no history of CAD and underwent CACS by multi-detector computed tomography (CT) were retrospectively enrolled. All subjects opted to have a CACS evaluation for screening purposes as part of a routine health examination.

We defined CACS percentiles using the KOICA registry as the Korean reference score, which is a large multicenter registry of a single-ethnicity (Korean) sample of 85,945 asymptomatic individuals who underwent a health examination at 5 health-care centers including the Seoul National University Hospital Healthcare System Gangnam Center between 2002 and 2014 [17]. We defined severe CAC as a CACS greater than the 90th percentile for their age and sex (5-year interval, from 20–24 to ≥75 years of age (S1 Table). Subjects with a CACS less than the 50th percentile for their age and sex were used as the control group. Individuals with CACS between the observed 50th and 90th percentile for age and sex were excluded. Of the original 2,188 subjects evaluated, 1,688 subjects were included (n = 327 in severe CAC group and n = 1,361 in control group). Genotype data were produced using the Korean Chip (K-CHIP) in 400 samples (n = 100 in severe CAC group and n = 300 in control group) based on the time of enrollment, and GWAS was conducted with this sample as the discovery set. The genome-wide significant SNP associated with severe CAC in the discovery set was screened using the TaqMan fluorogenic 5’ nuclease assay (ABI, Foster City, CA, USA) in the replication set (n = 227 in severe CAC group and n = 1,061 in control group).

### Ethics statement

The Institutional Review Board of the Seoul National University Hospital approved the storage of bio-specimens with informed consent (IRB no. 1103-127-357). We used the bio-specimens retrospectively. The board approved this study protocol (IRB no. 1504-017-662), and informed consent for research use of bio-specimens was waived by the board. The study was conducted in accordance with the Declaration of Helsinki.

### Hybridization on Affymetrix Axiom KORV1.0–96 array

Axiom2.0 Reagent Kit (Affymetrix, Santa Clara, CA, USA) was used according to the manufacturer’s protocol. About 200 ng of genomic DNA was amplified and randomly fragmented into 25 to 125 base pair (bp) fragments. Genomic DNA initial amplification was performed in 40 μl reaction volume, containing 20 μl volume of genomic DNA at a concentration of 10 ng/μl and 20 μl of Denaturation Master Mix. The reaction of initial amplification was performed for 10 min at room temperature. Subsequently, the incubated products were amplified with 130 μl of Axiom 2.0 Neutral Soln, 225 μl of Axiom 2.0 Amp Soln, and 5 μl of Axiom 2.0 Amp Enzyme. The amplification reactions were carried out for 23 h±1 h at 37°C. The amplification of products was performed under optimized reaction to amplify fragments between 200 and 1,100 bp. A fragmentation step then reduced the amplified products to segments of approximately 25–50 bp, which were then end-labeled using biotinylated nucleotides. Following hybridization, the bound target was washed under stringent conditions to remove non-specific background to minimize background noise caused by random ligation events. Each polymorphic nucleotide was queried via a multi-color ligation event carried out on the array surface. After ligation, the arrays were stained and imaged on the GeneTitan MC Instrument (Affymetrix, Santa Clara, CA, USA). The image was analyzed using Genotyping Console Software (Affymetrix, Santa Clara, CA, USA). Genotype data were produced using K-CHIP available through the K-CHIP consortium. K-CHIP was designed by the Center for Genome Science, Korea National Institute of Health, Korea (4845–301, 3000–3031).

### Genotyping and quality control

We performed systematic quality control steps on the raw genotype data and obtained 550,779 SNPs. SNPs with a minor allele frequency of case and control <1%, a call rate of case or control <95%. A significant deviation from Hardy–Weinberg equilibrium in controls (p<0.0001) were excluded. We also excluded SNPs likely to be false-positive associations due to wrong clustering via visual inspection of the cluster plots.

### TaqMan assay

The genotyping of SNP which met Bonferroni correction for genome-wide significance of severe CAC in the discovery set was screened using the TaqMan fluorogenic 5’ nuclease assay (ABI, Foster City, CA, USA) in the replication set. The final volume of polymerase chain reaction (PCR) was 5 ul, containing 10 ng of genomic DNA and 2.5 ul of TaqMan Universal PCR Master Mix, with 0.13 ul of 20X Assay Mix (Assay ID C___2684958_10). Thermal cycle conditions were as follows: 50°C for 2 min to activate the uracil N-glycosylase and to prevent carry-over contamination, 95xC for 10 min to activate the DNA polymerase, followed by 45 cycles of 95°C for 15 s and 60°C for 1 min. All PCR were performed using 384-well plates by a Dual 384-Well GeneAmp PCR System 9700 (ABI, Foster City, CA, USA), and the endpoint fluorescent readings were performed on an ABI PRISM 7900 HT Sequence Detection System (ABI, Foster City, CA, USA). Duplicate samples and negative controls were included to ensure accuracy of genotyping.

### Conventional risk factors

Baseline demographic characteristics, including age, sex, body weight, and height, were recorded. Body mass index (BMI) was calculated as weight divided by height squared in kg/m^2^. Information on risk factors and past medical history of smoking, hypertension, and diabetes mellitus was obtained using self-administered questionnaires. Presence of hypertension was defined as systolic/diastolic blood pressure >140/90 mmHg or a history of being diagnosed with hypertension or current treatment with antihypertensive medication.

Diabetes mellitus was defined by fasting blood glucose ≥7.0 mmol/l (≥126 mg/dl), hemoglobin A1c ≥6.5%, history of diabetes diagnosis, or taking medication for diabetes. Laboratory tests included lipid profiles, fasting glucose, and hemoglobin A1c.

### Cardiac CT and analysis for CACS

Coronary CT was performed using either a 256-slice multidetector CT scanner (Brilliance iCT 256; Philips Medical Systems, Cleveland, Ohio) or a 16-slice scanner (Somatom Sensation 16; Siemens Medical Solutions, Forchheim, Germany). A calcium scan was performed using standard prospective or retrospective methods with a 330- to 370-ms gantry rotation time. Image data were reconstructed using a 2.5–3 mm slice thickness. All scans were performed with electrocardiogram-gated dose modulation. The estimated radiation doses for CACS ranged between 0.7 and 1.5 mSv. The CACS was calculated quantitatively according to the method described by Agatston et al. [18] and a software program was used (Rapidia 2.8; INFINITT, Seoul, Republic of Korea).

### Statistics

We performed GWAS in the discovery set (n = 400) with a case–control study between the subjects with severe CAC (>90th percentile for age and sex) and control (≤50th percentile for age and sex). The results were verified using the validation test. The SNP determined to have genome-wide significance after Bonferroni correction in the discovery set was screened using the TaqMan fluorogenic 5’ nuclease assay (ABI, Foster City, CA, USA) in the replication set (n = 1288). A p-value less than 0.05 was considered significant in the replication set.

Logistical regression analyses were used to calculate the odds ratios (OR), 95% confidence intervals (CI), and corresponding p-values of each SNP of severe coronary calcification, controlling for age, sex, hypertension, and diabetes as covariates with additive models. All analyses were two-tailed, and p-values <0.05 were considered statistically significant. Statistical tests were performed using PLINK version 1.9 (https://www.cog-genomics.org/plink2; Free Software Foundation Inc., Boston, MA, USA). R statistical software package, version 3.1.1 (R development Core Team; R Foundation for Statistical Computing, Vienna, Austria) was used to analyze statistical data and to draw the Manhattan plot of -log10. In the regional plot, hg19/1000 Genomes Nov 2014 ASN was used as the reference panel for linkage disequilibrium [19].

## Results

### Study population characteristics

The characteristics of the study population including 400 subjects in the discovery set and 1,288 subjects in the replication set are described in Table 1. Between the two sets, the basal characteristics were not significantly different (p>0.05 in all parameters).

**Table 1:** Baseline characteristics of study population.

	Discovery set				Validation set			
	Total*	control	Severe CAC	p-value	Total*	control	Severe CAC	p-value
**N (%)**	400	300 (75.0)	100 (25.0)		1,288	1061 (82.4)	227 (17.6)	
**Male, %**	78.0	77.3	80.0	0.676	75.2	72.5	88.1	<0.001
**Age, mean±SD, years**	52.9**±**7.1	52.5**±**7.2	54.2**±**6.4	0.046	52.9**±**7.3	52.8**±**7.4	53.3**±**7.0	0.403
**CACS, mean±SD**	93.7**±**276.3	0.5**±**4.0	373.5**±**449.6	<0.001	64.2**±**265.9	0.4**±**3.1	362.2**±**542.5	<0.001
**CACS, median [IQ range]**	0 [0, 15.4]	0 [0, 0]	202.0 [93.0, 503.5]		0 [0, 0]	0 [0, 0]	213 [93.4, 409.9]	
**CACS, %**				<0.001				<0.001
** 0**	73.5	98.0	0		79.7	96.8	0	
** 0<CACS<100**	9.0	2.0	30.0		7.3	3.2	26.4	
** 100≤CACS<400**	10.3	0	41.0		8.2	0	46.7	
** CACS≥400**	7.3	0	29.0		4.7	0	26.9	
**BMI**	24.3**±**2.6	24.1**±**2.6	24.8**±**2.6	0.031	24.2**±**2.7	24.0**±**2.6	25.0**±**2.9	0.611
**Systolic blood pressure, mmHg**	117.3**±**13.4	116.5**±**13.4	119.6**±**13.1	0.054	117.5**±**13.6	117.0**±**13.8	119.5**±**12.8	0.017
**Diastolic blood pressure, mmHg**	77.8**±**9.9	77.2**±**9.8	79.8**±**10.2	0.027	78.0**±**10.4	77.6**±**10.7	80.3**±**8.8	<0.001
**Fasting blood glucose, mg/dl**	103.1**±**21.1	100.4**±**17.8	111.366**±**27.6	<0.001	101.0**±**18.2	99.8**±**16.8	106.9**±**22.8	<0.001
**Hemoglobin A1c, %**	5.8**±0**.6	5.8**±0**.6	6.1**±0**.8	0.001	5.8**±0**.6	5.7**±0**.5	6.0**±0**.8	<0.001
**Low-density lipoprotein cholesterol, mg/dl**	123.1**±**29.3	123.7**±**28.9	121.2**±**30.4	0.484	123.2**±**30.9	123.7**±**30.9	120.8**±**30.7	0.217
**High-density lipoprotein cholesterol, mg/dl**	52.0**±**12.6	52.0**±**12.7	52.1**±**12.1	0.909	52.2**±**12.4	52.7**±**12.1	49.8**±**13.9	0.002
**Triglycerides, mg/dl**	133.9**±**110.7	132.7**±**117.9	137.7**±**84.6	0.709	124.8**±**73.2	121.9**±**74.3	138.2**±**66.5	0.003
**C-reactive protein, mg/dl**	0.13**±0**.38	0.13**±0**.43	0.13**±0**.18	0.998	0.12**±0**.255	0.12**±0**.26	0.12**±**.19	0.847
**Hypertension, %**	29.3	23.7	46.0	<0.001	28.6	26.0	40.5	<0.001
**Diabetes, %**	14.0	10.0	26.0	<0.001	10.7	8.2	17.6	<0.001
**Current smoker, %**	19.0	18.0	21.8	0.431	17.8	17.0	21.5	0.127
**Ex-smoker, %**	37.7	35.6	43.7	0.200	38.0	34.8	53.1	<0.001

CACS, coronary artery calcium score; IQ, interquartile; BMI, body mass index

*all p-values > 0.05 in discovery set vs. validation set

Among the 400 subjects in the discovery set, 312 (78.0%) were men. The mean age was 52.9±7.1 years, and the median CACS was zero [interquartile range 0, 15.4]. Study participants in the severe CAC group were older and had slightly higher BMI, diastolic blood pressure, fasting glucose, and hemoglobin A1c and higher proportion of hypertension and diabetes. In the control group, 98% of subjects had zero calcium. As shown in S1 Table, CACS was classified as severe with respect to age and sex, and men <40 years old and women <55 years old with any CACS greater than 0 were classified as having severe CAC because individuals below the 90th percentile had a CACS of 0. Therefore, in these age groups, severe CAC indicates earlier CAC presence.

### GWAS of severe coronary calcification

**Fig 1:**
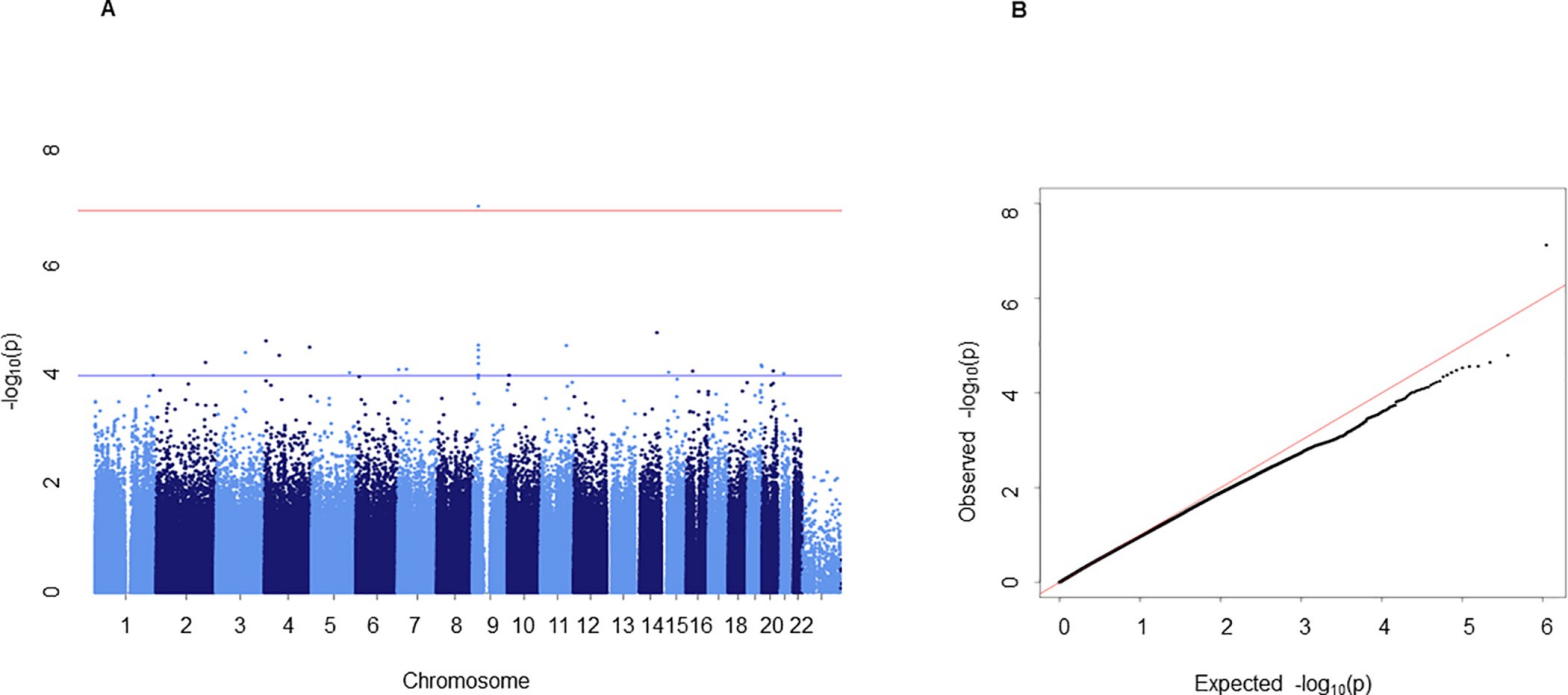
**Manhattan plot of genome-wide association signals with severe coronary artery calcification (A) and a quantile–quantile plot (B) in the discovery set.** In the Manhattan plot, the x-axis represents the SNP markers on each chromosome. The y-axis shows the -log10 p-value (logistic regression). The red horizontal line represents the genome-wide significance threshold p = 9.078E-08, and the blue horizontal line represents the genome-wide suggestiveness threshold p = 1E-04.

In the discovery set, an association analysis was conducted for severe CAC using 550,779 SNPs that passed the quality control. Quantile–quantile plots showed generally little evidence for statistic inflation and good agreement, except in the tail where deviations are expected due to genuine associations (Fig 1). Fig 1 also shows the Manhattan plot of the GWAS for cases with severe CAC.

We performed the GWAS in the discovery set after adjusting for age, sex, hypertension, and diabetes using the additive genetic model of logistic regression. One SNP (rs10757272) in the intronic region of the cyclin-dependent kinase inhibitor 2B anti-sense RNA gene (*CDKN2B-AS1*) in chromosome 9p21.3 passed Bonferroni correction (at a threshold of p = 9.08E-08) for genome-wide significance in the discovery set (p = 7.55E-08). Rs10757272 was also significant in the validation set by TaqMan assay (p = 0.036).

**Fig 2:**
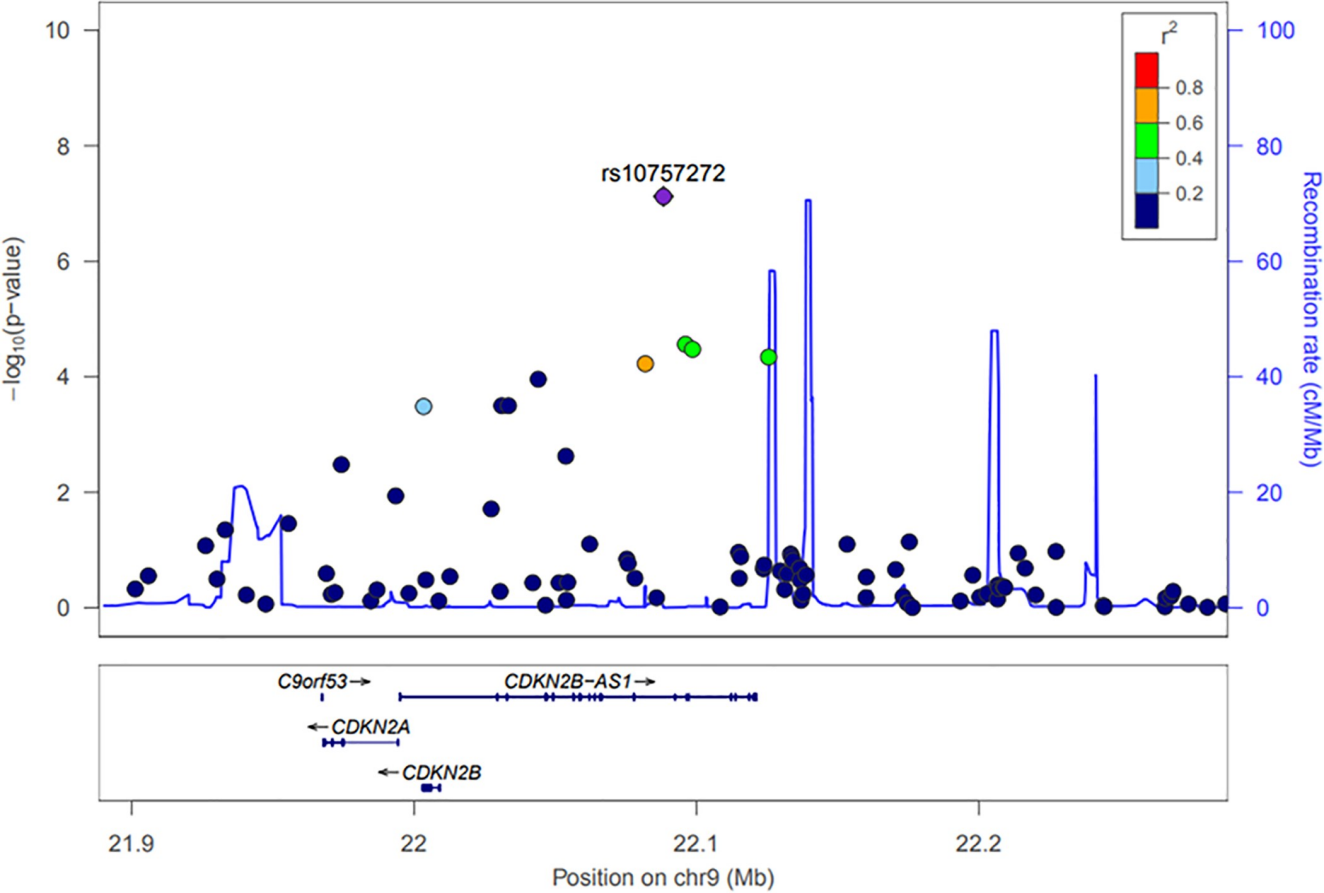
Regional association plot in chromosomes 9. The purple diamonds indicate the associated SNP according to joint analyses. Nearby SNPs are color-coded according to the level of linkage disequilibrium with the top SNP. The left y-axis shows the significance of the association on a -log10 p-value (logistic regression), and the right y-axis shows the recombination rate across the region. Estimated recombination rates from the 1000 Genomes Project Asian base data and hg19 database [19] are plotted with the blue line to reflect the local linkage disequilibrium structure.

The regional plot in chromosome 9 is provided in Fig 2, which shows rs10757272 as the top SNP and nearby SNPs with the level of linkage disequilibrium with rs10757272.

The subjects with rs10757272 were found to have an increased OR of having severe CAC in multivariate logistic regression analysis after adjusting for age, sex, hypertension, and diabetes (adjusted OR 3.24 and 95% CI 2.11–4.97) (Table 2).

**Table 2:** SNP associated with severe coronary calcification in Koreans and the odds ratios.

SNP	Chr	Position*	Risk allele	Nearest gene	Allele frequency		HWE	OR†	95% CI	p-value	
					Case	Control				Discovery set	Replication set
rs10757272	9	22088260	T	*CDKN2B-AS1*	0.785	0.582	0.95	3.24	2.11–4.97	7.55E-08	0.036

SNP, single-nucleotide polymorphism; Chr, chromosome number; HWE, Hardy–Weinberg equilibrium; OR, odds ratio; CI, confidence interval.

*The SNP positions are indexed to the National Center for Biotechnology Information build 37.

^†^Association results generated from additive model of logistic regression analyses adjusted with age, sex, hypertension, and diabetes

## Discussion

In this community-based population without CAD, we demonstrated that one SNP (rs10757272) in the chromosome 9p21.3 locus reached significance for severe CAC for age and sex in Koreans. We also replicated the association of this SNP in subjects in the validation set. Therefore, 9p21.3 variant promoted coronary artery calcification in subclinical state among asymptomatic Koreans. It has mainly been previously reported in the Western population, implying trans-ancestry risk for CAC.

Recently, the National Human Genome Research Institute GWAS catalog shows that >50 SNPs for coronary artery susceptibility variants were identified during the GWAS era, pooling >100,000 subjects of European descent [4]. In multiple cohorts of European descent, other SNPs on chromosome 9p21.3 have been consistently associated with CAD (rs10757274 and rs2383206), and MI (rs2383207 and rs10757278) [5–9]. In a study with Korean patients who had >70% luminal narrowing in at least one vessel by coronary angiography, percutaneous coronary angioplasty, coronary artery bypass graft, or MI, four SNPs (rs10757274, rs2383206, rs2383207, and rs10757278) on chromosome 9p21.3 was reported as an important susceptibility locus [20]. A collaborative meta-analysis reported an association between chromosome 9p21.3 risk locus and a greater burden of angiographically defined CAD in many races including Korean, Chinese, and Japanese [21]. However, it had no association with MI in the presence of background CAD. They hypothesized that the 9p21.3 risk locus primarily precipitates the development of atherosclerotic lesion and significant coronary stenosis rather than promoting plaque rupture or thrombosis. A recent study has reported that variation in 9p21.3 may not only be an independent genetic risk factor for CAD, but may also modify the association between diastolic blood pressure levels and the extent of subclinical coronary atherosclerosis [22].

SNPs in this region on chromosome 9p21.3 also have been associated with the quantity of CAC. A meta-analysis reported genome-wide significant associations with CACS >100 versus <100 for SNP rs1333049 on chromosome 9p21.3 mainly in White populations in Northern Europe and North America [10]. Gong et al. reported that the rs1333049 allele in 9p21.3 was associated with the extent or severity of CAC in CAD patients and increased the risk of CV events in the presence of CAC. Therefore, they suggested that the 9p21.3 locus related calcification may affect the susceptibility to CAD [23].

We used reference values for CACS defined according to age and sex in Koreans. In men <40 years old and women <55 years old, the presence of CAC (CAC> 0) was defined as severe CAC, in contrast to the control individuals, who had zero calcium. Therefore, in these age groups, we suggested that SNP rs10757272 promotes the presence of CAC in the population of asymptomatic Koreans without prior history of CAD. CAC is a prognostic factor in asymptomatic subjects as well as CAD patients. Prior asymptomatic population-based studies have documented the relationship between higher CAC and worsened cardiovascular prognosis [24, 25]. A sub-group study of CONFIRM in patients without known CAD demonstrated that patients without luminal narrowing but with CAC experienced greater risk of 5-year mortality than those with zero calcium [26].

However, in GWAS, most of the loci associated with CAD are not related to the risk factors for atherosclerosis. The mechanisms of gene-disease association remain undefined [4]. Even if the suggestive loci in our study have no direct association with known traditional risk factor for CAD, some biological studies have suggested an atherosclerotic mechanism of chromosome 9p21.3 loci. Chromosome 9p21.3 resides in a region distant from known coding regions, with tumor suppressor genes of cyclin-dependent kinase inhibitors (*CDKN2A* and *CDKN2B*) and methylthioadenosine phosphorylase. The nearest genes to the 9p21.3 region are *CDKN2A* and *CDKN2B*, which are associated with cell cycling and death [27] and relevant to the atherosclerotic process with excessive vascular smooth muscle cell proliferation [28] and proliferation of neointimal content of macrophages [29]. In addition, rs10757272 in the intronic region of *CDKN2B-AS1* was reported to be associated with platelet reactivity [30]. These are key pathological mechanisms promoting early development and progression of atherosclerosis. Further studies are necessary to investigate the pathogenesis of atherosclerosis and vascular calcification and clinical utility of genes in 9p21.3 including *CDKN2A* and *CDKN2B*.

This study has limitations. First, its sample size was small. The discovery and validation sets were different sets, but drawn from the same population in a single center. A validation study with a different Korean population should be performed in the future. Second, the study population was composed of subjects who underwent regular health check-ups. Thus, this study should be performed using a larger set of clinical CAD patients with MI or coronary revascularization. This study has some strong points. This is the first GWAS study showing the association of CAC quantity in an asymptomatic community-based Korean population. Because Koreans are ideal for case-control association studies as they are ethnically and genetically homogeneous populations, all study subjects were of the same ethnicity (Korean). Moreover, we used a more precise definition of severe CAC. Age is the strongest independent factor for CAC and men and women show a different pattern of increment in CACS. CACS increases with age, and it increases rapidly after age 50 in Korean men and after age 60 in Korean women, with a “time-lag” of about 10 years in women [31]. Therefore, in spite of the absolute CACS, we used the CACS above the 90th percentile of the healthy reference sample in each age and sex in Koreans as a relative cut-off value of severe CAC.

## Conclusions

In asymptomatic community-based Korean populations, SNP rs10757272 on chromosome 9p21.3 were associated with severe CAC based on each age and sex. Therefore, it was associated with promoting coronary calcification in subclinical state. These data could support the role of genetic variation as contributors to CAC in Koreans. Subsequent independent replication in large sample size and functional studies including patients with subclinical and clinical CAD are required to elucidate its genetic basis and the molecular pathways underlying the pathogenesis of CAC and CAD.

## Supporting information

S1 Table CACS in control group and severe CAC group in each group as stratified by age and sex. (DOCX)
