# Supplementary material for: Genome-wide association study of coronary artery calcification in asymptomatic Korean populations
Source: PLoS One. 2019 Mar 28;14(3):e0214370. doi: 10.1371/journal.pone.0214370 (PMC6438465; doi:10.1371/journal.pone.0214370)
Supplement: S1 Table — (DOCX) [file pone.0214370.s001.docx]

Supplementary Table 1

| **years** | **CACS in control group*** | | **CACS in severe CAC group†** | |
| --- | --- | --- | --- | --- |
|  | **Male** | **Female** | **Male** | **Female** |
| 30-34 | 0 | 0 | > 0 | > 0 |
| 35-39 | 0 | 0 | > 0 | > 0 |
| 40-44 | 0 | 0 | > 4.0 | > 0 |
| 45-49 | 0 | 0 | > 28.0 | > 0 |
| 50-54 | 0 | 0 | > 77.9 | > 0 |
| 55-59 | 0 | 0 | > 159.3 | > 19.0 |
| 60-64 | ≤7 | 0 | > 272.5 | > 75.3 |
| 65-69 | ≤28 | 0 | > 484.0 | > 165.9 |
| 70-74 | ≤52.36 | ≤12.85 | > 591 | > 327.1 |
| ≥75 | ≤107.65 | ≤49.4 | > 1077.0 | > 400.0 |

CAC, coronary artery calcification; CACS, coronary artery calcium score. *CACS less than the observed 50th percentile and †CACS greater than the observed 90th percentile of CACS across the age in each sex group using the KOICA registry (N=85,945) [14].
